# Cholera: a comparison of the 2008-9 and 2010 Outbreaks in Kadoma City, Zimbabwe

**DOI:** 10.11604/pamj.2015.20.221.5197

**Published:** 2015-03-11

**Authors:** Brian Abel Maponga, Daniel Chirundu, Notion Tafara Gombe, Mufuta Tshimanga, Donewell Bangure, Lucia Takundwa

**Affiliations:** 1 Department of Community Medicine, University of Zimbabwe, Zimbabwe; 2 Department of Health, Kadoma City Council, Kadoma, Zimbabwe

**Keywords:** Cholera trends, Kadoma, Zimbabwe

## Abstract

**Introduction:**

Kadoma City experienced cholera outbreaks in 2008-9, and 2010, affecting 6,393 and 123 people, respectively. A study was conducted to compare epidemiology of the cholera outbreaks.

**Methods:**

A descriptive cross sectional study was conducted, analyzing line list data for the 2 outbreaks. Proportions, means were generated and compared using the Chi Square test at 5% level of significance.

**Results:**

Cholera cases were similar by gender and age, with the 20-30 years group being most affected. Rimuka township contributed 80% and 100% of city cases in 2008-9 and 2010, respectively, p value = 0.000. In 2008-9, 91% of cholera cases presented within 2 days compared to 98% in 2010. Delay seeking treatment increased from 58% to 73% (p value = 0.001), with gender, and place equally affected. The 2010 outbreak evolved faster, resulting in higher proportion being managed in CTU. CFR was 2% in 2008-9, and 3.3% in 2010 (p value =0.31).

**Conclusion:**

The 2008-9 and 2010 cholera outbreaks were similar by age and gender. Rimuka Township was most affected by the outbreaks. There was worsening of delay seeking treatment. The 2010 outbreak was more rapid, leading to early opening of CTC. CFR was consistently above 1%.

## Introduction

Cholera is a highly infectious disease that is characterized by profuse watery diarrhea, with high case fatalities if untreated [1]. In Zimbabwe, cholera is a notifiable disease under the Public Health Act (15:09) [2]. According to the International Health Regulations (WHO 2005), member countries are expected to report any cholera cases to the World Health Organization [3]. Cholera is an acute disease caused by a gram negative bacterium Vibrio Cholerae either serogroup 01 or serogroup 0139 [4]. If treatment is delayed or inadequate, death from dehydration and circulatory collapse may result. If not treated case fatality may be as high as 50% but with appropriate treatment it can drop to less than 1%. The mainstay of treatment is rehydration orally or intravenously. The extremely short incubation period of 2 hours to five days enhances the potentially explosive pattern of outbreaks as the number of cases can rise sharply [4].

Between 1995 and 2009, Africa has been contributing above 90% of global cholera cases, but the proportion reversed to below 90% due to the large outbreak that occurred in Haiti in late 2010 [5–7]. Zimbabwe contributed more than half of African cholera cases between 2008 and 2009, and a decline in cholera in 2010 saw the contribution declining to less than 2%. Zimbabwe experienced a serious cholera outbreak in 2008 and 2009. The outbreak started in Chitungwiza and spread to 55 out of the 62 districts, infecting 99,704 people, killing 4,420 people. Between November 2008 to May 2009, Kadoma City attended to 6,393 (6.4%) of the 99,704 national cholera cases and had 123 (2.8%) of the 4,420 national deaths. Kadoma City experienced another cholera outbreak during the first half of 2010, which had 127 cholera cases and 4 deaths. This is against the background that Kadoma City, being urban, contributes 0.6% of the national population. During the two cholera outbreaks, Kadoma City Health Department maintained line lists, both on a paper based system, and Epi Info^™^ based system. The line lists for each outbreak were meant to primarily describe patients‘ demographic data, clinical presentation, clinical management, and treatment outcome. The study was conducted because the city experienced back to back cholera outbreaks, despite interventions which were put in place during and after the outbreaks. Interventions are thought to have affected the hosts (including behavior), the environment and to a lesser extent the infectious agent. Therefore, a comparison of the epidemiology of cholera during the two outbreaks was done.

## Methods

A Descriptive Cross Sectional study was conducted using secondary analysis of data for the 2008-9 and 2010 cholera outbreaks. The outbreak line lists were already in Epi Info^™^. The data was cleaned for errors that occurred during entry. Epi Info^™^ was used to generate and compare proportions. Chi Square tests were done, at 5% level of significance. Permission was obtained from Kadoma City Council and, and the Health Studies Office in Zimbabwe.

## Results

**Figure 1:**
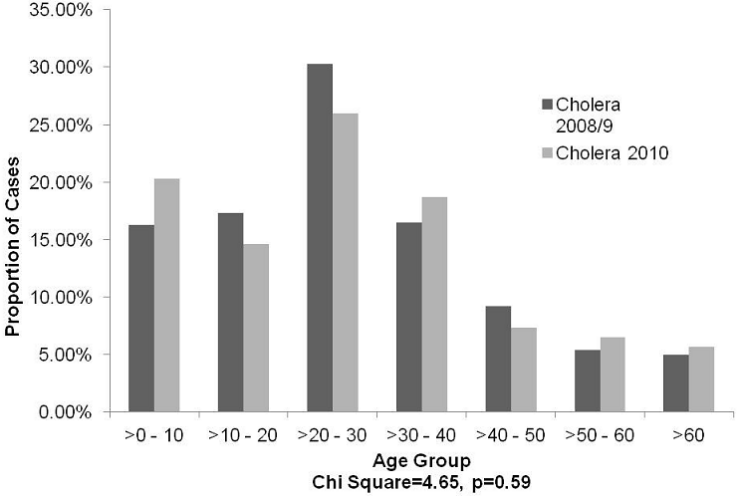
Age Distribution of Cholera Cases, Kadoma City, 2008-9 and 2012, Kadoma City, Zimbabwe

The 2008-9 outbreak had 6,393 cholera cases entered, with 42,717 entries being done out of a possible 44,751 entries for 7 selected variables, translating to 95% completeness. In 2010, 123 cholera cases were entered, with 851 entries out of the expected 861 entries for the same 7 selected variables, translating to 98.8% completeness. The proportion of missing entries for the variable “treatment outcome“ was highest, 29%, in 2008-9, compared to less than 6% in the 2010 outbreak, p = 0.001. All the entries that were missing for both outbreaks were for discharged patients, whilst all deaths were captured. However, some variables which were included in the 2010 outbreak were not in the 2008-9 outbreak line lists, and were not compared. These included data on symptoms, hydration status, treatment plan, and laboratory results. Table 1 shows the comparison of cholera cases by age, gender, and place. There was no statistically significant difference between males and females, p = 0.20. The median age of the cholera cases was similar for the two outbreaks, being 27 and 26 years respectively. During the 2008-9 outbreaks, 4.7% of the cases were under two years, compared to 5.7% in 2010, p = 0.59. Further analysis of the 2010 data indicated that all three laboratory tests on children below two years were negative. There was a similar age distribution for the two outbreaks (p = 0.59). The 20-30 years age group being most affected, as shown in Figure 1. The cholera cases differed significantly in terms of place of residence, with 89% of cases being Kadoma City residents in 2008-09, compared to 99% in 2010, p = 0.002. For the Kadoma City residents, 80% of the cases were Rimuka Residents in 2008-9, compared to 100% in 2010, p = 0.000. The outbreaks differed significantly in terms of duration, lasting 193 days in 2008-9, and 45 days in 2010.

**Table 1:** Comparison of cholera cases by person and place, Kadoma City, Zimbabwe, 2008-9 and 2010

Attribute	Frequency (%)		Chi Square	p value
	2008-9 Outbreak	2010 Outbreak		
**Age**				
Mean (s.d.)	28 (16.7)	27 (17.7)	0.43	0.51
Median (Q_1_=, Q_3_=)	27 (18; 37)	26 (14;36)		
Range (Min; Max)	0; 99	0; 78		
< 2 years	295 (4.7)	7 (5.7)	4.65	0.59
< 5 years	529 (8.4)	16 (13)	4.38	0.11
Female	3084 (48.3)	67 (54.5)	1.61	0.20
Male	3303 (51.7)	56 (45.5)		
**Kadoma Resident:**				
Yes	5679(89)	121(99)	9.76	0.002
No	688(11)	2(1)		
Rimuka Township	4561(80.3)	121(100)	28.3	0.000
Other Township	1118(19.7)	0		

### Duration of symptoms

**Figure 2:**
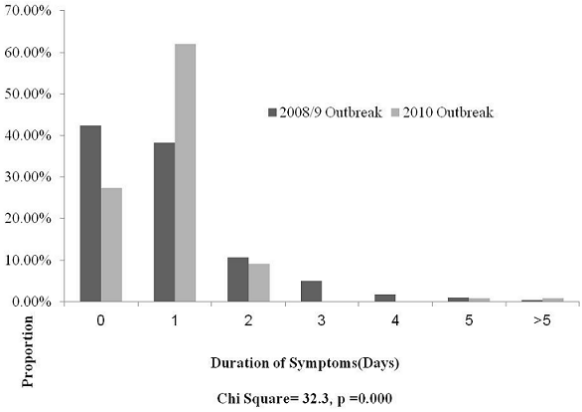
Duration of Symptoms for Cholera Cases, Kadoma City, Zimbabwe, 2008-9 and 2010

Figure 2 shows the distribution of duration of symptoms for the 2008-9 and 2010 cholera outbreaks. The mode number of days of symptoms was zero days for the 2008-9 outbreak, and one day for the 2010 outbreak. About 97% of cholera cases presented within 2 days of onset of symptoms, in 2010, compared to 91% in 2008-9, p = 0.000.

#### Delay seeking treatment

Delay seeking treatment was defined as presenting at treatment unit after the day of onset of symptoms of cholera. Table 2 shows the comparison of delay in seeking treatment, for the 2008-9 and 2010 outbreaks. The overall delay increased significantly from 58% to 73%, p value= 0.001. Both the females (p = 0.021) and males (p = 0.03) showed similar significant increases in the proportions delaying seeking treatment. The proportion of cholera cases that delayed seeking treatment increased across all the age groups, but the increase was statistically significant in the above 15 years age group (0.003).

**Table 2:** Delay in seeking treatment, 2008-9 and 2010 cholera outbreaks, Kadoma City, Zimbabwe

Category	2008-9 Outbreak (%)	2010 Outbreak (%)	Chi Square	p value
**Delayed:**				
Yes	3649(58)	88(73)	10.46	0.001
No	2680(42)	33(27)		
**Males Delayed:**				
Yes	1880(58)	40(73)	4.55	0.033
No	1391(42)	15(27)		
**Female Delayed:**				
Yes	1768(58)	48(73)	5.29	0.021
No	1289(42)	18(27)		
**< 5 years Delayed:**				
Yes	285(54)	11(73)	1.45	0.23
No	241(46)	4(27)		
**5-14 years Delayed:**				
Yes	365(51)	10(59)	0.45	0.71
No	348(49)	7(41)		
**>15 years Delayed:**				
Yes	2968(59)	67(75)	8.88	0.003
No	2058(41)	22(25)		
**City Resident Delayed:**				
Yes	3267(58)	86(72)	9.11	0.025
No	2360(42)	33(28)		
**Non City Resident Delayed:**				
Yes	373(55)	2		
No	312(45)	0	Not Valid	0.50
**Rimuka Resident Delayed:**				
Yes	2618(58)	86(72)	9.29	0.002
No	1905(42)	33(28)		

### Cholera case management and mortality

There were 14 Oral Rehydration Points (ORP), 2 Cholera Treatment Centres (CTC) and 1 Cholera Treatment Unit (CTU) in 2008-9, compared to 5 ORPs, 1 CTC and 1 CTU in 2010 outbreak. The proportion of cholera cases that were managed at the ORPs only was similar for the two outbreaks, p = 0.82. The case management differed in terms of cases managed at CTC and CTU, p = 0.000. It took 33 cases to be seen at the hospital CTU to warrant opening of CTC in Rimuka, compared to 64 in 2008-9. A total of 127 deaths out of the 6,393 reported cholera cases occurred during the 2008-9 outbreaks, translating to a CFR of 2.0%. Four deaths out of 123 cases, giving a CFR of 3.3% occurred in the 2010 outbreak. The increase in CFR was not statistically significant, p = 0.31. CFR could not be compared by person, place and time, as there were only 4 deaths in 2010.

## Discussion

The study was conducted to compare the 2008-9 and 2010 cholera outbreaks. A comparison of the epidemiology of the cholera outbreaks, influence of gender, age, and place of residence on health seeking behavior, management of cases, and the mortality amongst the cases was done. The 2008-9 and 2010 cholera line lists had data of good quality, with the majority, more than 95% of entries having been completed. The city health department should be commended for embracing the use of information technology in public health interventions. The data, being of good quality, just shows how much dedication was given, despite the pressure of work during cholera outbreaks. The two cholera outbreaks showed similar trends in terms of person (age, and gender). The 20-30 years age group bore the biggest burden. Similarly in Harare in 2007 and in 2008 the cholera cases had a median age of 28 years [8]. An outbreak of cholera in Burundi in 1994 had more than half the cases (54%) being above the 15 years age group [9]. In Mexico between 1991 and 2002, the most affected age group was the 25 to 44 years age group [10]. This trend could suggest the impact cholera will have on economic productivity, as the economically active age groups are affected. Thus, long term investments, such as was done in Mexico, in water and sanitation, that reduce cholera occurrence could reduce economic losses due to cholera [10]. However, contrasting trends were noted in an Indian study in 1992-4 where the 1-9 years age group was the most affected over the three years 1992-4 [11]. In Iran, between 1997 and 2002, the less than five years age group had the highest proportion of positive cholera results (26%) [12]. During the 2008-9 outbreaks, 4.7% of the cases were under two years, compared to 5.7% in 2010. Further analysis of the 2010 data indicated that all three laboratory tests on children below two years were negative, suggesting that the cases might not have been cholera at all, thus in keeping with the cholera definition of cholera that excludes children under 2 years [1].

The two outbreaks differed significantly in terms of place, as the 2008-9 cholera outbreak was more widespread in the city, and beyond the borders of the city. Conversely, the 2010 outbreak was more confined, with the cases being concentrated in Rimuka Township. Repeated outbreaks in Rimuka Township may suggest environmental contamination, whilst an improvement of water and sanitation conditions in some areas could have resulted in decline in cases. Similarly, a cholera outbreak in Burundi‘s Rumonge town affected all streets, with attack rates increasing with closer proximity to Lake Tanganyika, the possible source of contamination [9]. The 2008-9 and 2010 outbreaks differed significantly in terms of duration, being 193 and 45 days, respectively. The significant variation could be explained by the onset of the 2008-9 outbreaks during the start of the 2008 rain season, thus persisted throughout the season, whilst the 2010 outbreak started towards the end of the rainy season, March 2010. Even though there are no laboratory results readily available for the 2008-9 outbreaks, assuming that 96% of positive cholera cases presented within 2 days, it could be assumed that the 5774 (91%) cases that presented within 2 days could be true cholera cases. This is despite there being no outbreak of cholera had been reported in Kadoma City prior to the 2008-9 outbreaks, and the majority of the health workers were not conversant with cholera. Improvements in diagnosis could be attributed to the case management trainings conducted during the 2008-9 and 2010 outbreaks. It could also be safely assumed that in future, cases of watery diarrhea that present with more than two days symptoms might not be cholera after all. The proportion of cholera cases that delayed seeking treatment significantly increased from 58% to 73%. This increase in proportions that delayed seeking treatment did not differ by place and gender, but differed for the different age groups. The increase was significant in the above 15 years age group only. The implication is that the longer the patients delayed seeking treatment the higher the chances of environmental contamination, increasing the risk of transmission to contacts. At the same time, the longer the delay, the higher the risk of dying from dehydration and electrolyte imbalance. This could explain the fact that the CFR remained above the WHO acceptable CFR of 1% for the two outbreaks, despite the different interventions put in place before, during and after the outbreaks such as health education [1]. This could lead us to question the effectiveness of health education methods.

It took 33 cases (2010) and 64 cases (2008-9) to be seen in the CTU to necessitate the opening of a CTC in Rimuka Township, the epicenter of the outbreak. For a CTC to be opened there has to be at least ten patients requiring care in the CTU, at the same time. This suggest that the outbreak in 2010 was more rapid compared to 2008-9, at the same time short. This could explain the higher proportion of cases, 25% in 2010, compared to 1% in 2008-9 that were managed in the CTU. The CTU, at Kadoma Hospital, is about 10km from Rimuka suburb, the epicenter of the 2008-9 and 2010 outbreak, which could have resulted in patients having to travel further, increasing dehydration, and risk of death. In Harare, the high CFR of 3.98% could have been attributed to the fact that the cholera cases were being managed at two treatment units in Budiriro and Beatrice Infectious Diseases Hospital [8]. The four cholera deaths in 2010, made it impossible to compare the mortality in terms of person, place and time, for the 2008-9 and 2010 outbreaks. Some variables were not captured during the 2008-9 outbreaks, such as laboratory results, symptoms, hydration and treatment plan, thus could not be analyzed. Some of the variables could have explained some of the findings such as mortality above 1%.

## Conclusion

The 2008-9 and 2010 cholera outbreaks were similar in terms of age and sex distribution. There were significant differences in place of residence, with the 2008-9 being more widespread in the city and beyond, whilst the 2010 outbreak was more concentrated in Rimuka Township. The bulk of the cholera cases, more than 90%, presented within 2 days of onset of symptoms. There was an increase in the proportion of cholera cases that delayed seeking treatment for the two outbreaks. Utilization of ORPs remained the same for the two outbreaks. Outbreak progressed faster in 2010, compared to the 2008-9 outbreaks, leading to early opening of CTC. Mortality was consistently above the 1% WHO recommended threshold for the two outbreaks.
